# NUAK2: an emerging acral melanoma oncogene

**DOI:** 10.18632/oncotarget.325

**Published:** 2011-09-10

**Authors:** Takeshi Namiki, Sergio G. Coelho, Vincent J. Hearing

**Affiliations:** ^1^Laboratory of Cell Biology, National Cancer Institute, National Institutes of Health, Bethesda, MD 20814, USA; ^2^Department of Dermatology, Yokohama Minato Red Cross Hospital, Yokohama, Kanagawa 231-0801, Japan

**Keywords:** NUAK2, acral melanoma, migration, metastasis, oncogene

## Abstract

Recent technological advances in cancer genomics make it possible to dissect complicated genomic aberrations of melanomas. In particular, several specific genomic aberrations including 11q13 amplification and KIT aberrations have been identified in acral melanomas. We recently identified NUAK2 at 1q32 as a promising oncogene in acral melanomas and reported its significant roles in tumorigenesis in melanoma cells using both *in vitro* and *in vivo* analyses. NUAK2 as a member of the AMPK family has several intriguing aspects both as an oncogene and as a tumor suppressor gene. Here we review genomic aberrations of melanomas focusing on acral melanomas to emphasize the possible roles of NUAK2 in tumorigenesis in general and suggest that NUAK2 has pivotal roles in acral melanomagenesis.

## INTRODUCTION

The identification of genes that participate in oncogenesis has facilitated recent development of molecular targeted therapies against cancer (reviewed in [1-3]). In the past several years, the identification of mutations in the BRAF gene in melanomas has led to the development of molecular therapies targeting those mutations in metastatic melanomas, which is a highly lethal disease [4-6]. However, recent studies have also shown that the frequency of mutation of the BRAF gene in melanomas has clear discrepancies depending on the subtype of melanomas [7-11]. A high frequency of aberrations in the BRAF gene is observed in Non-CSD (CSD:chronic sun-induced damage) melanomas [8, 12], while the BRAF V599E mutation has a relatively low frequency in other subtypes of melanomas, including frequencies ranging from 15% to 33% in acral melanomas, which occur on the palms, soles and subungual sites [8, 10], but almost 0% in mucosal melanomas [9, 10]. These discrepancies suggest the hypothesis that other genomic aberrations may play important roles in melanomagenesis in subtypes of melanomas other than Non-CSD melanomas and that further explorations of genomic aberrations, which can be ideal and effective targets for molecular targeted therapies against melanomas, are required to develop more effective and alternative therapies targeting a wide range of melanomas. In acral melanomas, several genomic aberrations, including amplification of 11q13 and KIT mutations, have been found [13-15]. The elucidation of genomic aberrations in melanomas using detailed analyses of public array-CGH databases suggests that genomic gain at chromosome 1q32 has profound effects on acral melanomagenesis. NUAK2, a gene at this locus, has regulatory impacts on the proliferation and migration of melanoma cells [16].

The SNF1/AMP-activated protein kinase (AMPK) family functions to control the balance of cellular metabolisms, and is activated by the cellular AMP:ATP ratio that is regulated by metabolic stresses such as hypoxia and glucose deprivation [17, 18]. AMPK is composed of 3 subunits (α, β and γ), and the α-subunit is the catalytic subunit. This catalytic subunit family includes 5 members: AMPK-α1, AMPK-α2 [19-21], MELK [22], NUAK2/SNARK [23, 24] and NUAK1/ARK5 [25]. Twelve protein kinases (BRSK1, BRSK2, NUAK1, NUAK2, QIK, QSK, SIK, MARK1, MARK2, MARK3, MARK4 and MELK) have been identified as AMPK-α1 and AMPK-α2 related kinases in the human kinome [26, 27]. The function of each member of this catalytic subunit are also closely connected to tumor formation and metastasis [18, 28, 29]. However, those functions participating in tumor formation and metastasis are different depending on each subunit. As shown in previous studies, AMPK-α1 and AMPK-α2 have anti-oncogenic properties [30-32] while ARK5 has pro-oncogenic properties [33]. The exact function(s) and mechanism(s) of NUAK2 (also known as SNARK), the fourth member of catalytic subunit of AMPK, remain unknown. NUAK2 has been speculated to have contradictory functions on tumorigenesis as a tumor suppressor or as an oncogene [24, 34]. In this review, we discuss the oncogenic role of NUAK2 and its clinical significance in melanoma patients, as well as its regulation by intracellular signaling pathways including LKB1 and CaMKKβ in melanoma cells.

## ACRAL MELANOMA ONCOGENES AND ITS CLINICAL ASPECTS

**Figure 1:**
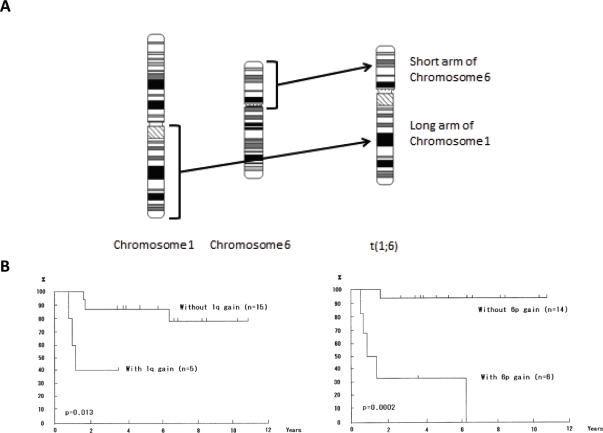
Chromosome rearrangements and impact on survival of melanoma patients A) Translocations in melanoma cells. The long arm of chromosome 1 is translocated to the short arm of chromosome 6 to result in the t(1;6) chromosome. B) Metaphase CGH analyses revealed that both chromosomal gains of 1q and 6p significantly correlate with the survival of melanoma patients (ref. 62). Kaplan-Meier survival analyses for overall survival are shown. *P* values are indicated.

Recent tenacious efforts by investigators have accelerated the elucidation of genomic alterations in melanoma cells. The first step to decipher enigmas in complex genomic alterations in melanoma cells had started by taking advantage of techniques that had been utilized to elucidate chromosomal abnormalities in hematopoietic malignancies [35-39]. Those techniques, which examine structural and numerical chromosomal aberrations in hematopoietic malignancies and sarcomas, had elucidated a reciprocal translocation between the long arms of chromosomes 9 and 22 (e.g., the Philadelphia chromosome) in chronic myelogenous leukemia (CML) [40-41] and resulted in identifying the oncogenic fusion protein of BCR-Abl [42-44]. Those discoveries eventually led to the development of a BCR-Abl inhibitor (imatinib mesylate; Gleevec), which improved the management of patients who suffer from CML [45-48]. As this example has clearly indicated, the identification of oncogenes and tumor suppressor genes, which can be guided by the characterization of structural and numerical aberrations of chromosomes in cancer cells, is an important step and an efficient approach to develop effective therapeutic modalities to control lethal diseases and improve the quality of life of cancer patients. For malignant melanoma, early studies pointed out nonrandom chromosomal aberrations involving chromosomes 1, 6, 7, 10 and 19 [35-39], and those aberrations had been confirmed by a larger number of cases [49]. A series of those early studies emphasizes that chromosomal aberrations involving chromosomes 1 and 6 are important in melanomagenesis, and particularly a recurring translocation of t(1;6) was noted (Fig. 1a) [50]. However, progress in the elucidation of oncogenes and tumor suppressor genes in melanomas by cytogenetic analyses were hampered technologically due to difficulties in obtaining metaphase preparations that are suitable for karyotyping from primary tumors. Comparative genomic hybridization (CGH) had overcome those technical obstacles by using genomic DNA as source materials to analyze genomic alterations of primary tumors [51], and microarray technologies extensively increased the resolutions of CGH analyses [52]. Those novel technologies were swiftly applied to analyses of genomic alterations of melanoma cells [13, 53]. Analyses of a large number of cases with different clinical subtypes of primary melanomas using array-CGH revealed that melanomas have four different subtypes from a genomic point of view: melanomas on skin with chronic sun-induced damage (CSD melanoma), melanomas on skin without chronic sun-induced damage (Non-CSD melanoma), Acral melanomas and Mucosal melanomas [54]. Those novel findings obtained by a series of CGH studies in addition to mutation analyses including the BRAF gene enabled progress in the research fields of molecular pathogenesis of melanomas in contrast to the previous concept, which was speculated from clinical and histopathological observations, that melanomas do not have enough evidence to subcategorize them into different subsets [55].

Acral melanomas occur on nailbeds and plantar regions such as the palms of the hands and soles of the feet [56]. The histopathological expression of acral melanomas is not always acral lentiginous melanoma, and other histopathological types of superficial spreading melanoma and nodular melanoma also exist [57]. Although the aggressiveness of acral melanomas had been disputed due to small sample size [58], recent data with a large cohort supports the aggressiveness of acral melanomas [59]. CGH analyses have shown unique genomic changes of acral melanomas that differ from those of other melanomas. At the chromosomal level, numbers of genomic aberrations in the whole genome were higher in acral melanomas. Particularly numbers of amplifications are significantly high, and amplifications at 11q13 and 5p15 are noted in acral melanomas [60, 61]. Those changes were also verified by array-CGH analyses [53]. High resolution mapping of an amplicon at 11q13-14 in breast cancer suggests CCND1, S6K2 and GAB2 as candidate genes in that region [62]. Fluorescence in situ hybridization (FISH) and/or immunohistochemical analyses showed that amplification and high expression of CCND1 were also observed in acral melanomas [63, 64]. Interestingly, the amplification of CCND1 is relatively rare in melanomas with BRAF-NRAS mutations and may have similar effects on melanoma cell growth as the activation of the mitogen-activated protein kinase (MAPK) signaling pathway resulting from BRAF and/or NRAS mutations [65, 66]. Another array-CGH study indicated the importance of the 4q12 region, where a narrow amplification is observed in acral, mucosal and CSD melanomas and the KIT gene resides at that locus [67]. Activation of KIT results from amplification and/or mutation, and mutations are frequently observed in exons 11, 13 and 17 [15, 68]. This KIT activation is intriguing, because it could be a direct target of therapies against acral melanomas using inhibitors targeting KIT (such as imatinib mesylate) [69, 70]. A particularly important point in the tumorigenesis of acral melanomas that is different from other subtypes of melanomas such as Non-CSD melanomas is that BRAF mutations occur at lower frequencies in acral melanomas [71]. These observations imply that other genetic aberrations may have a profound effects on the tumorigenesis of acral melanomas.

**Figure 2:**
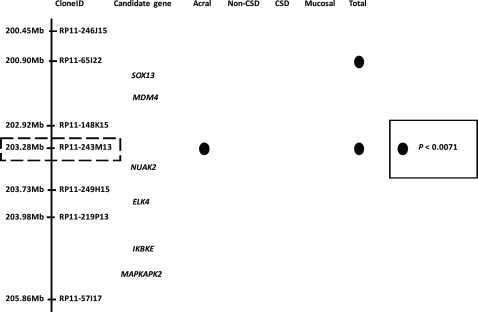
Candidate genes at the 1q32 locus Analyses of a public array-CGH database indicated that a gene around clone RP11-243M13 is the most promising candidate oncogene at this locus. *NUAK2* resides at the vicinity of this clone. Filled circles represent genomic clones with *P* values <0.0071 in each subset of melanoma. Adapted from [16].

CGH combined with analyses of clinical parameters are a powerful approach to identify genomic loci that have significant impact on the clinical outcome of melanoma patients. Both the classical cytogenetic approach and the CGH approach share the same implication that the long arm of chromosome 1 and the short arm of chromosome 6 may have a profound effect on the tumorigenesis of melanomas since both chromosomal regions have a significant impact on the clinical outcome of patient survival (Fig.1b) [50, 61]. Statistical analyses comparing tumor thickness, which is a predominant factor for clinical outcome in primary melanomas, by taking advantage of public array-CGH databases have revealed that 1q32, among the 4 loci of 1q21-23, 1q32, 6p23-25 and 6p21, is significantly correlated with tumor thickness in acral melanomas. Using the candidate gene approach, NUAK2 at this locus has been revealed as a promising gene that participates in the clinical outcome of acral melanomas (Fig. 2) [16].

## PHYSIOLOGICAL ROLES OF NUAK2 AND THE AMPK FAMILY

NUAK2 is the fourth member of the AMPK family of kinases and shares a similar catalytic domain of the sucrose-non-fermenting protein kinase (SNF1)/AMP-activated protein kinase (AMPK) family of serine/threonine protein kinases. The NUAK2 gene resides at 1q32 and encodes 630 amino acid residues that are translated into a protein of approximately 76 kDa [23]. In general, AMPK family members are made up as heterotrimeric complexes of a catalytic α-subunit and regulatory β- and γ-subunits, and they act as an energy sensor to monitor energy homeostasis by binding AMP to the γ-subunit [17]. The binding of AMP to the γ-subunit stimulates the kinase activity of the α-subunit and promotes the phosphorylation of a Thr residue in the kinase domain, which results in the boost of kinase activity by additive (and/or synergic) effects of both stimulations [72]. Upstream regulators of the AMPK family have been identified including LKB1 and the calmodulin-dependent protein kinase kinases (CaMKKα and CaMKKβ) [73, 74]. LKB1 regulates 13 AMPK related kinases including NUAK2 as an upstream regulator [26]. Downstream effects of the AMPK family are diverse. The main effects of the AMPK family are functions relating to cellular metabolisms including the regulation of glucose intake [75]. The AMPK family has also diverse effects via regulation of the transcription of various genes related to the control of cell proliferation and cell polarity [76, 77]. Some of these effects are suspected to be tissue specific [17].

The amount of knowledge about the regulation and function(s) of NUAK2 is quite limited compared to AMPK-α1 and AMPK-α2. However, several important functions related to myosin filaments and cytoskeleton organization have been revealed. Myosin phosphatase target subunit 1 (MYPT1) was identified as a specific substrate for NUAK2. MYPT1 is phosphorylated by NUAK2 at sites other than Thr696 and The853, which are known as Rho-kinase (ROCK) phosphorylation sites [78]. Further, a study showed that unc-82, which encodes a serine/threonine kinase orthologous to human NUAK1/NUAK2 (ARK5/SNARK) in *Caenorhabditis elegans*, participates in maintaining the integrity of components of myosin filaments. Disruption of unc-82 by mutations causes defects in cytoskeleton reorganization during embryogenesis [79]. Another study showed that NUAK2 is able to associate with myosin phosphatase Rho-interacting protein (MRIP) and this association results in increased levels of myosin regulatory light chain (MLC) phosphorylation and facilitates the formation of stress fibers. Activities resulting from those associations of NUAK2 and MRIP are independent of NUAK2 kinase activity and those associations inhibit fiber disassembly and MYPT1-mediated MLC dephosphorylation. Important roles of NUAK2 on fiber maintenance in proliferating cells and the existence of a positive –feedback loop regulating actin stress fibers independent of the MLC kinase Rho-associated protein kinase (ROCK) have been indicated [80]. As the AMPK family of kinases in general functions as a sensor of metabolic homeostasis in cells, NUAK2 is activated by cellular stresses such as glucose deprivation, rotenone and sorbitol [23, 81]. Muscle contraction increases glucose transport by increasing NUAK2 activity in skeletal muscle cells, which suggests that NUAK2 functions to connect to the cytoskeleton modulation induced by muscle contraction with energy homeostasis in cells [82]. Those results indicate that NUAK2 plays a pivotal role in regulating the cytoskeleton, which is also important in cell proliferation and motility, and suggests that NUAK2 connects metabolic homeostasis and cell motility. The disruption of those mechanisms in normal cells may reflect cell proliferation and migration in cancer cells.

## POTENTIAL ONCOGENIC ROLES OF NUAK2 AND ITS SIGNIFICANCE IN ACRAL MELANOMAS

Disruption of the normal regulation and function(s) of NUAK2 may lead to the dysregulation of proliferation and migration in cancer cells. However, the exact effects and mechanisms participating in tumorigenesis remain to be elucidated due to the lack of sufficient molecular studies of NUAK2. Several conflicting results on tumorigenesis including cell proliferation, apoptosis and migration have been reported. The knockdown of NUAK2 using siRNA or shRNA reduces cell proliferation *in vitro* and tumor growth *in vivo* in melanomas, and the extent of those reductions of cell proliferation varies depending on the different genomic aberrations of melanoma cells [16]. Another study with a carcinogen (azoxymethane) induced colorectal tumorigenesis model using NUAK2-deficient mice has shown that hemiallelic loss of NUAK2 contributes to carcinogen-induced neoplastic and preneoplastic lesions of colorectal carcinomas, which suggests there are tumor suppressive roles of NUAK2 in the early phase of tumorigenesis and suggests the minor effects of NUAK2 deficiency on cell proliferation *in vivo* from the profile of the proliferating cell population [83]. Over-expression of NUAK2 induced prolongation of cell survival of HepG2 cells in nutrient-deprived circumstances [24]. This function of NUAK2 is partly dependent on anti-apoptotic properties against apoptosis induced by death ligand such as the CD95 ligand, TRAIL and TNF-α which shows that NUAK2 is a kinase induced by TNF-α [78, 84]. However, NUAK2 functions during apoptosis are different depending on the melanoma cell lines [16]. NUAK2 also has effects on the migration of cancer cells as speculated from studies on myosin filaments and cytoskeleton organization in normal cells. An initial study revealed that the over-expression of NUAK2 has effects on cell-cell detachment in glucose deprived circumstances and suggested that over-expression of NUAK2 induced dysregulation of mechanisms to maintain the cytoskeleton and to coordinate its attachment to the cell membrane [24]. CD95 stimulation facilitates cell motility and invasiveness of MCF7-FB cells, which up-regulates NUAK2 expression by stimulation of the CD95 ligand [84]. That evidence suggests that the effects of NUAK2 on tumorigenesis are different depending on the tissue and the phase of tumorigenesis, and that NUAK2 participates in increased cell motility and invasiveness.

**Figure 3:**
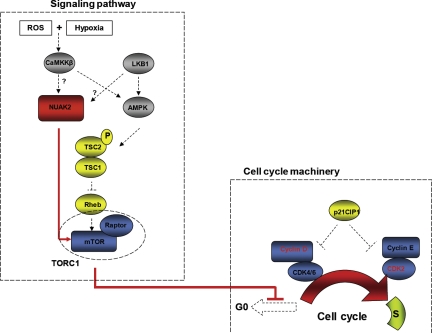
Hypothetical schematic of the regulation of NUAK2 in acral melanomas Hypothetically, both reactive oxygen species (ROS) and hypoxia activate NUAK2 through CaMKKβ in acral melanoma cells, and result in dysregulation of cell cycle machinery through the mTOR pathway. Hypothetical pathways, which are activated in acral melanoma cells, are depicted as red line and/or arrows.

UV irradiation is one of the major causes of cutaneous melanomas, but acral and mucosal melanomas are protected from exposure to UV irradiation due to their anatomical locations. Thus, the molecular pathogenesis of acral and mucosal melanomas should be different from that of cutaneous melanomas arising from sun-exposed areas such as Non-CSD melanomas, and causes other than UV irradiation, such as reactive oxygen species (ROS), may play an important role in the melanomagenesis of acral and/or mucosal melanomas. The HGF/SF transgenic mouse model is prone to develop cutaneous melanomas following UV irradiation [85]. In that mouse model, LKB1 is one of the major downstream targets and uncoupling of the LKB1-AMPK pathway by oncogenic BRAF is one possible mechanism to promote the proliferation of melanoma cells with BRAF mutations [86]. Another study has substantiated the pivotal role of the LKB1-AMPK pathway in melanomagenesis [87]. The anatomical distribution of lentigines in Peutz-Jeghers syndrome, which is caused by mutations in the LKB1 gene, is almost identical to the distribution of both acral and mucosal melanomas. Although those observations imply that the LKB1-AMPK pathway may also play a role in the neoplastic formation of melanocytes distributed in those areas, melanomas are relatively rare with a few exceptions in those areas of patients with Peutz-Jeghers syndrome [88, 89]. Recent studies have shown that both ROS and hypoxia can activate AMPK through calcium release-activated calcium (CRAC) channels and CaMKKβ independent of LKB1 [90, 91]. Melanocytes are speculated to reside both in ROS abundant and in hypoxic conditions from observations that ROS are constantly generated as a byproduct of melanin synthesis in melanocytes [92], and that the epidermis where melanocytes reside is a relatively hypoxic environment (with oxygen levels ranging from 1.5% to 5.0%) [93]. Although the exact mechanism(s) that connects ROS and/or hypoxia to NUAK2 are still under investigation, those mechanisms should be further elucidated to explain melanomagenesis arising from acral areas. The downstream pathways by which NUAK2 regulates the cell cycle machinery have been examined with knockdown experiments of NUAK2 by siRNA and suggest that NUAK2 regulates Cyclin D1 and Cyclin D3 expression through the mTOR pathway to control cell proliferation (Fig. 3) [16]. The mTOR pathway participates in controlling progression through the cell cycle. Several studies using melanoma cells suggest that the mTOR pathway also participates in controlling the balance between senescence and quiescence under oncogenic and/or tumor suppressive stimuli. These intricate molecular mechanisms may partly explain the dual functions of NUAK2 as an oncogene and as a tumor suppressor gene [94, 95]. Clinical data also suggest that NUAK2 plays a pivotal role in melanomagenesis in acral areas and has effects on the survival of patients with acral melanomas. Univariate analysis using Kaplan-Meier curves showed that high expression of NUAK2 has an impact on relapse-free survival of acral melanoma patients (*P* = 0.0036), and multivariate analysis using multiple Cox regression analysis also showed the impact of NUAK2 on relapse-free survival (hazard ratio = 3.88, 95% confidence interval = 1.44-10.50, *P* = 0.0075). Interestingly, those impacts of NUAK2 on melanoma patient survival are significant at relapse-free survival of acral melanomas compared to weak significances at Non-CSD melanomas and overall survival. Those observations lead to the speculation that NUAK2 has more profound effects on cell migration resulting in worsening relapse-free survival of acral melanoma patients as suggested by both a migration assay and a wound healing assay [16].

## FUTURE PERSPECTIVES FOR THERAPEUTIC IMPLICATIONS

The recent identification of KIT mutations in acral, mucosal and CSD melanomas led to the development of therapeutic modalities using imatinib mesylate to target KIT activation. Although initial studies achieved only poor responses using imatinib mesylate against melanomas, the stratification and appropriate selection of patients based on KIT activation by mutations and amplifications have improved responses of melanoma patients to imatinib mesylate [70, 98]. As this example has clearly shown, the use of molecular targeting agents should be based on a better understanding of molecular carcinogenesis and an appropriate selection of patients [96, 97]. In acral melanomas, the frequency of KIT mutations is relatively low and therapeutic strategies targeting other mutations and amplifications are required to improve overall efficacy and response taking advantage of inhibitors targeting those genetic aberrations. A better understanding of the roles of AMPK family members including NUAK2 in acral melanomagenesis should be a necessary step to improve the management of acral melanoma patients.
